# Local government expenditure centralization and spatial variation in working-age mortality

**DOI:** 10.1016/j.ssmph.2025.101791

**Published:** 2025-03-27

**Authors:** Rourke O'Brien, Manuel Schechtl, Robert Manduca, Atheendar Venkataramani

**Affiliations:** a Yale University, United States; b UNC-Chapel Hill, United States; c University of Michigan, United States; d University of Pennsylvania, United States

## Abstract

Research finds disparities in local government spending to be one driver of place-based variation in population health outcomes in the U.S. This study asks: net of the amount of local government spending, does the *centralization* of local government expenditures shape spatial variation in working age mortality? We find that in more centralized local fiscal structures, that is, where the county government performs relatively more of the total local government spending, there is less cross-census tract variation in midlife mortality. In doing so, we reveal how the structure of local government—inherited from history and largely outside the purview of politics and policy discussion—impacts place-based variation of population health outcomes.

## Highlights

• There is substantial variation in rates of working age mortality across census tracts within U.S. counties.
• Counties where government spending is more centralized exhibit less spatial variation in working age mortality across census tracts.
• The fiscal structure of subnational governments is an important determinant of spatial variation in population health.

## 1 Introduction

Population health outcomes, including working-age mortality, vary markedly across the United States (Boing et al., 2020; Dwyer-Lindgren et al., 2017; Montez et al., 2020). This spatial inequality in health parallels a broader body of social science research showing that exposure to place influences a wide range of outcomes across the life course, including educational attainment, employment opportunities, and the likelihood of incarceration (Chetty et al., 2016; Sharkey & Faber, 2014). Scholars often attribute such place-based disparities to differences in state and local government policy, particularly the intensity of public spending. These differences are of particularly consequence in the U.S. context, where subnational governments play a key role in financing services that directly shape social and population health outcomes. Indeed, the degree to which states and localities invest in social spending—for everything from safety net programs to public health spending to basic education—can result in stark variations in health and well-being from one community to another (see, e.g., Mays & Smith, 2011; Lobao & Hooks, 2003; Kim & Jurey, 2013).

Unlike other advanced democracies, such as the UK and France, where national governments predominantly fund and implement public programs, the United States delegates much of this responsibility to subnational governments (see Kim, Lotz, and Blöchliger, 2013). Consequently, key interventions affecting population health—such as public health infrastructure, K–12 and higher education, and social safety net programs like Medicaid and Unemployment Insurance—depend heavily on state and local funding (see Sparer, 2020; see also Rodden, 2006). This decentralized system, coupled with significant autonomy for state and local governments, produces notable disparities in both the design of public policies and the amount of government spending across jurisdictions. These inequalities in spending and program implementation help explain why health outcomes can differ so sharply between communities, even within the same state. Examining these structural and fiscal differences is essential for understanding the uneven geography of health and the persistence of working-age mortality disparities nationwide.

Recent studies underscore the importance of state-level policy in driving spatial variation in population health (see, e.g., Goldstein et al., 2020; Bradley et al., 2016; see also Rubin et al., 2016). For example, Montez and colleagues (2020) have demonstrated that more generous or liberal state policies—including expanded Medicaid access, stronger public health investments, and robust social welfare programs—are associated with better health outcomes, including lower mortality. Their findings point to the critical role that states play in shaping health outcomes, given their extensive policymaking authority. Yet state-level policies cannot account for the notable variation in health observed at more local levels, underscoring the need for finer-grained analyses of how the structure and function of local governments impact spatial variation in health.

A key challenge in studying the effect of local, i.e., sub-state, government spending on population health outcomes is the sheer complexity and heterogeneity of local governance in the United States. As of 2017, there are more than 90,000 local government entities in the U.S., including cities, towns, townships, school districts, and special taxing districts, each with distinct taxing authorities, fiscal responsibilities, and public spending mandates (U.S. Census of Governments 2017). The relative role of each of these jurisdictions in generating revenue and funding public services varies widely among—and even within—the fifty states. This heterogeneity in how local services are financed and delivered-–including which government(s) is fiscally responsible for each category of spending and service delivery—impedes efforts to examine how local government spending impacts population health outcomes.

Given this complexity, a common empirical strategy is to aggregate all local government spending within a county, regardless of which specific jurisdiction performed the spending; this yields roughly comparable measures of total government expenditures in a “county area”. McCullough and Leider (2016) used this approach to estimate the association between local government expenditures and population health across U.S. counties. Consistent with expectations, they found that counties with higher levels of overall (aggregated) local government spending had on average better health outcomes, including longer life expectancy. They went on to examine the impact of different categories of expenditure, and found investments in public health, K-12 education, fire protection, corrections, libraries, and housing development were all associated with improvements in overall health rankings.

McCollough and Leider provide strong evidence that local government expenditures can partially explain observed variation in health outcomes across U.S. counties. Yet detailed mortality data reveal that the vast majority of place-based variation in life expectancy is not between counties but within: Boing and colleagues (2020) found that 70 % of the spatial variation in life expectancy is between census tracts within the same county, while only 11 % of the variation occurs between counties and 19 % between states. In extreme cases, the difference in life expectancy at birth between the lowest- and highest-performing census tracts within the same county can exceed 40 years. This suggests that examining average health outcomes at the county-level, while an improvement over state-level analyses, still obscures much of the place-based variation in working age mortality.

This study aims to test whether and how the structure of local government may be contributing to observed variation in population health outcomes across census tracts within a county. We take a novel approach in the population health literature by examining not the amount of local government spending but instead whether the source of local government spending impacts the spatial patterning of health outcomes. Specifically, we investigate whether counties that are more fiscally centralized, i.e., where a greater share of total expenditures is performed by the county government versus by constituent cities or towns or districts, exhibit less overall spatial variation in working-age mortality. Although there are alternative ways scholars of public administration operationalize centralization—e.g., the centralization of own source revenues or of public employment (see, e.g., Rodden, 2004; Panizza, 1999)—in this study we focus on the centralization of government expenditures given extant work linking government expenditures to population health outcomes. By focusing on this structural dimension of local finance, we extend the conversation beyond absolute spending levels to consider how the configuration of local governance can help explain health disparities at the sub-county level.

We hypothesize there will be less spatial variation in working age mortality across census tracts in counties where relatively more of the total local government spending is performed by the county government, which typically sits at the highest level of spatial aggregation. Put simply, we hypothesize that in more centralized fiscal systems there will be less place-based variation in the quality or intensity of local government and therefore less place-based variation in social outcomes. Note that the centralization of local government spending is distinct from the amount of government spending; that is, centralized local governments can be characterized by high or low amounts of per capita spending. Therefore, we do not expect the overall mortality rate to necessarily be lower in more centralized local government structures; rather, we hypothesize there will be less place-based difference or overall variation in mortality rates across census tracts.

There are several reasons we might expect that greater fiscal centralization may yield less spatial variation in working-age mortality. First, fiscal centralization allows for a more coordinated and targeted allocation of resources within a county; county governments can pool resources and redistribute them more equitably across the entire geographic area, ensuring that under-resourced communities are not left behind (Prud'homme, 1995; Rodden, 2006). Second, centralized fiscal structures can mitigate the inequalities that often arise from fragmented local governance. In more decentralized systems, smaller local jurisdictions may have vastly different fiscal capacities (i.e., taxable wealth), leading to uneven levels of public investment; wealthier areas are able to fund high-quality healthcare, education, and social programs, while poorer areas struggle to provide even basic services (Fisher, 2016; Kim, Lotz, and Blöchliger, 2013). By consolidating fiscal responsibility at the county level, wealth disparities between local jurisdictions can be smoothed out, leading to more uniform levels of service provision across the county and reducing within-county inequalities in health outcomes (Prud'homme, 1995).

Third, more centralized fiscal systems benefit from economies of scale and more professionalized bureaucracies, which can lead to more efficient and effective delivery of public services ((Bahl & Linn, 1992; Oates, 1972). Larger, more centralized government entities may be better equipped to identify public health needs and implement interventions at a broader scale, leading to improved outcomes across different areas within the county (Rodden, 2006). In this sense, centralization not only equalizes resources and service provision but also enhances administrative capacity. Taken together, these dynamics suggest that greater fiscal centralization could dampen spatial disparities in mortality.

Most prior research examines fiscal centralization at the level of nation-states, with a focus on understanding how more or less centralized fiscal systems impact economic growth and shape economic inequality between regions (see, e.g., Neyapti, 2006). In the U.S. context, prior work has examined how local government *fragmentation*—often operationalized as the number of general-purpose governments (e.g., cities or towns), school districts, and special districts in a metropolitan area—impacts social outcomes. For example, using data from the 1997 Census of Governments, Hutson et al. (2012) find that more fragmented metropolitan areas exhibit greater racial health disparities, operationalized as the Black-White mortality ratio. A related study by Kim and Bruckner (2016) examining the 1970s and 1980s finds that counties with more local government fragmentation exhibit higher mortality among both White and Black Americans. These studies provide suggestive evidence that the structure of local government, beyond the level of spending, may be a key driver of differences in population health outcomes across metropolitan areas. Yet they tell us little about how the degree of fragmentation shapes the spatial patterning of health outcomes *within* metropolitan areas.

Centralization, by contrast, captures the extent to which government spending in each area is centralized, regardless of the number of local jurisdictions (see Boyne, 1992). Although areas with more jurisdictions tend to be, on average, less fiscally centralized, this is not necessarily or always the case; areas characterized by a high degree of jurisdictional fragmentation may have very centralized or very decentralized local government spending. In the analyses below we adjust for measures of local government fragmentation to isolate the net effect of spending centralization from the number of local governments. We also differ in our outcome of interest, focusing not on average outcomes in a county area but instead the overall degree of spatial variation in health—i.e., extent to which mortality outcomes are more or less similar are across census tracts—in a county area.

To date only one study has explored how the centralization of U.S. state and local governments impacts social outcomes, finding more centralized fiscal systems exhibit less spatial variation in the intergenerational economic mobility outcomes of low-income children (O’Brien et al., 2025). This suggests that, like fiscal redistribution—where higher level governments provide direct revenue support to poor areas to equalize resources, e.g., to fund K-12 education—fiscal centralization may another strategy to reduce spatial or place-based inequalities in the U.S. In this study we examine this potential impact on population health outcomes, focusing on working-age mortality which has increased markedly in recent decades. Given prior research finds working-age mortality to be sensitive to state and local government policy, we hypothesize that in more centralized local government structures, there will be less variation in mortality rates across census-tracts.

## 2 Study data and methods

### 2.1 Data

We estimate spatial variation in mortality using data from the Small Area Life Expectancy (USALEEP) project, which provides census-tract level estimates of age-specific mortality in 10-year age categories for deaths observed between the years 2011–2015 (Arias et al., 2018). Deaths from the National Vital Statistics Systems were linked to census tract using decedents’ residential address; data exist for 48 states (excluding Maine and Wisconsin) and nearly 90 % of U.S. census tracts. We focus on working age mortality, i.e., between the ages of 25 and 64; this permits us to capture place-based exposure where individuals live during their working years, after completing formal education and prior to migration in retirement. We calculate a Gini index to capture the total amount of variation in the age-specific mortality rate across census tracts within a county: a higher value corresponds to more dispersion in working age mortality whereas a lower value corresponds to more uniform outcomes. Appendix Table 1 and Appendix Figs. 1–4 reveal substantial heterogeneity in the degree of spatial variation in mortality across counties both within and between states and across counties of different size populations.

We measure local government expenditure centralization using data from the U.S. Census of Governments which includes a complete accounting of revenues and expenditures for more than 90,000 local government entities in the United States. These data are collected every 5-years (years ending in 2 and 7). To construct a measure of total government spending in a county area, we sum all local government expenditures within a county—including those of the county government, as well as any cities, towns, townships, and school districts. We exclude special districts due to data constraints that make it difficult to allocate their spending precisely to county areas, although results are not sensitive to this omission (see Appendix Fig. 6).

We then construct our measure of county expenditure centralization by taking the fraction of total local government spending in a county area performed by the county government. Expenditure data are from 2007, which is the wave immediately preceding our mortality observation period. We note, however, that fiscal centralization is a relatively stable characteristic of local government structure. Appendix Fig. 5 shows county area fiscal centralization changed little between 1977 and 2007 despite a substantial rise in income inequality between households and between places over that period; this reflects that centralization is a structural feature of local government that remains largely unaffected by changes in demography, economy or politics.

Data for the social and demographic control variables (detailed below) are taken from the 2010 U.S. Census.

### 2.2 Analytic approach

We examined the relationship between county area expenditure centralization and the Gini measuring within-county cross-census-tract spatial variation in working age mortality through a series of linear models estimated by OLS. For each age 10-year group, we first estimate a multivariable model (Model 1) that adjusts for fiscal and spatial covariates including the number of local governments, number of school districts, and number of census tracts in the county area, as well as the total county population and the total land area in square miles, all log-transformed. We then estimate Model 2 which includes total per capita government expenditure in the county area as a covariate to isolate spending centralization from spending amount. Finally, Model 3 introduces a tranche of social and demographic characteristics: income per capita, poverty rate, net migration rate, percentage Black residents, percent college graduates and percent over the age of 65 in addition to measures of the spatial variation in poverty, household income, and percentage Black residents across census tracts in the county.

We include state fixed effects in all models to restrict comparisons to counties within the same state. We do so for several reasons. For one, state government policy is an important driver of population health outcomes. Moreover, the structure of government finance varies substantially across states: for example, in Hawaii and West Virginia the state government performs nearly three-quarters of all state and local government spending whereas in other states, like Nebraska, local governments perform the majority of total spending. This variation thwarts efforts to meaningfully compare local governments across state lines, even after aggregating to comparable geographies (e.g., county area). We therefore also cluster standard errors at the state level given the fiscal interdependence of governments within the same state.

## 3 Study results

**Fig. 1:**
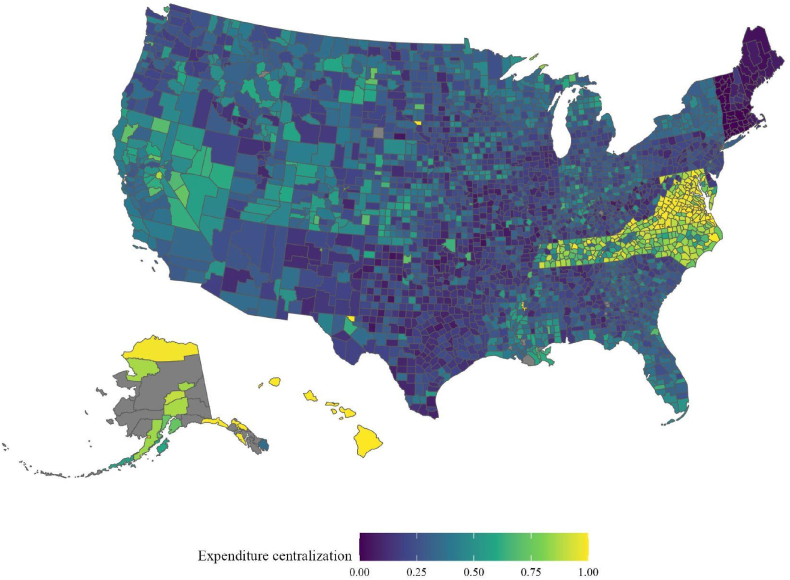
Local government expenditure centralization, U.S. County Areas (Authors' Calculations from U.S. Census Bureau, 2007).

Fig. 1 displays local government expenditure centralization across the 2783 counties in our analytic sample. There is substantial variation in the extent to which local government spending is centralized, ranging from a low of 0 % in Connecticut (which does not have county government) to a high more than 90 % for consolidated city-counties such as Baltimore City, San Francisco, and Nashville (Davidson County). Notably, centralization does not map onto contemporary differences in the political, economic or demographic character of state or local areas within or between states as the structure of U.S. subnational governments is the result of place-specific, path-dependent history (Béland & Lecours, 2014; Coen-Pirani & Wooley, 2018; O’Brien et al., 2025). For example, the relatively centralized structure of local government in Tennessee can be traced to its late 19th century founding by settlers moving West from Virginia and North Carolina where strong county government had been the norm throughout the colonial era. Tennessee was founded as a collection of counties, and the centrality of counties to fiscal governance would be codified in the state constitution and reified over the ensuing centuries in statute and fiscal practice (Laksa 1975, 1990; see O’Brien et al., 2025).

**Fig. 2:**
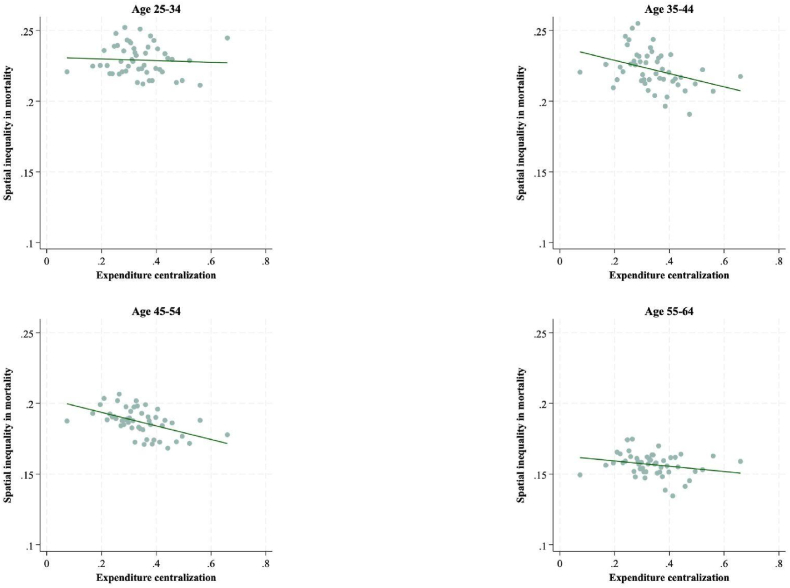
Local government expenditure centralization and spatial variation in working age mortality (Model 1).

Fig. 2 plots the relationship between local government expenditure centralization and spatial variation in working age mortality for each 10-year age group as estimated in Model 1; each dot in this binscatter plot corresponds to 50 county observations. Here we see a clear negative relationship between expenditure centralization and spatial variation in mortality for those aged 35–44 and 45-54—more centralized local government spending yields less place-based variation in mortality outcomes. Notably the association is less pronounced for the younger (25–34) and older (55–64) working age groups, perhaps due to the higher rates of migration (and therefore less consistent exposure to places) in earlier and later working years. (Full regression results displayed in appendix Tables 2–5).

**Fig. 3:**
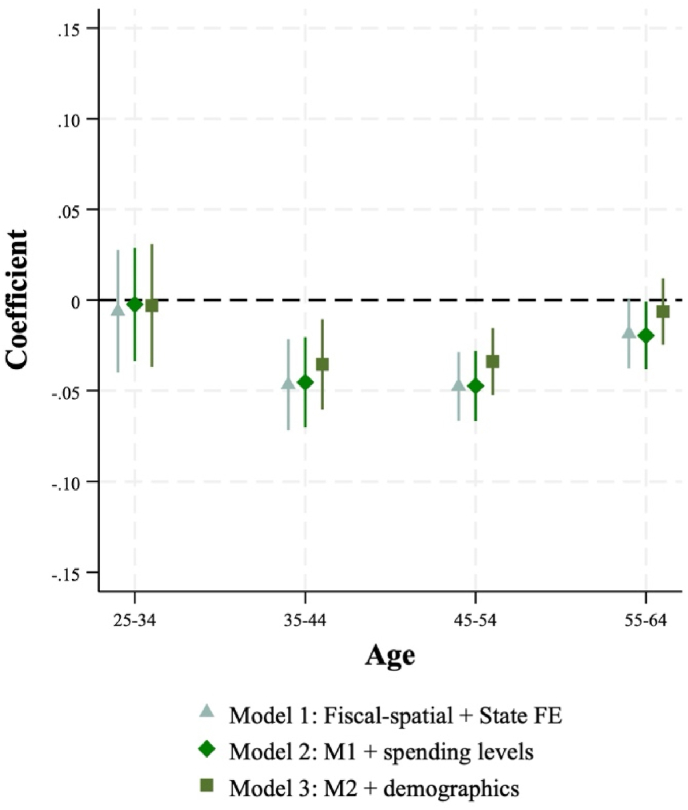
Coefficients from models estimating relationship between local government expenditure centralization and spatial variation in mortality by 10-year age groups.

Fig. 3 plots the estimated coefficient on county area expenditure centralization across each model specification and for each age group. Note that after including covariates in the model, the estimated coefficient on expenditure centralization becomes indistinguishable from zero for the youngest (aged 25–34) and oldest (aged 55–64) age groups. However, we continue to see a robust, negative association between the degree of county area expenditure centralization and the degree of spatial variation in mortality among those aged 35–44 and 45–54. The estimated coefficient indicates a 10-percentage point increase in expenditure centralization is associated with 0.4 percentage points less spatial variation in mortality among those aged 35–44 and a 0.3 percentage point reduction among those aged 45–54. Put another way, an increase from the centralization of Middlesex County, Connecticut (0 %) to that of Davidson County (Nashville), Tennessee (>90 %) would be associated with a 3 to 4 percentage point decrease in spatial variation in mortality for these age groups.

Point estimates reported in the appendix table indicate the estimated association between expenditure centralization and spatial variation in mortality is comparable to the estimate for spatial variation in household poverty. Notably, we find no evidence of an association between the level of (per capita) public expenditures in the county area and spatial variation in working age mortality; this provides further evidence that the structure or location of local government spending can impact the spatial patterning of health outcomes independent of the amount of spending. At the same time, we estimate a large, positive association between the overall level of income inequality in a county area and the degree of spatial variation in mortality outcomes across all working age groups.

In the online appendix we report findings from several robustness checks. First, we show our findings are unchanged by including the overall age-specific mortality rate as a model covariate (see Appendix Tables 6–9). Second, we show our findings are the same if we weight counties by their total population (see Appendix Tables 10–13). Third, we show our findings are substantively unchanged if we exclude school district spending; this suggests our findings are not driven by the structure of K-12 education finance (see Appendix Tables 14–17).

## 4 Limitations

One major limitation of this study is that we cannot explore patterns in working age mortality separately for males and females or for specific racial and ethnic groups; this is unfortunate as mortality levels and trends vary substantially and systematically across these population subgroups. Although we do adjust for the racial composition of local areas to ensure our findings are not confounded by the spatial patterning of residential segregation, future work should explore whether the spatial-variation-reducing impact of expenditure centralization differs across these groups. Another limitation of this study is that heterogeneity in the expenditure roles of local government entities inhibits our ability to examine which *types* of spending are more consequential for reducing spatial variation when centralized: are the associations we find driven by variation in spending on health or that on social safety net programs? A related limitation is that our measure of county-level expenditure centralization does not capture the considerable variation in the amount and centralization of state government spending. We account for this variation in our use of state fixed effects in the analyses above but are not able to study it directly.

Moving forward, scholars of health and place must build a data frame capable of detailing all forms of government spending in each area regardless of the source government. This will permit more systematic analyses of how the amount, source *and* type of government spending shapes health outcomes and contributes to place-based disparities. Finally, we note that while our results are consistent with work showing local government spending has a causal impact on health (e.g., Mays & Smith, 2011), our approach does not definitively establish a causal effect of centralization of spending on spatial variation in health outcomes.

## 5 Discussion & conclusion

This study finds that more centralized local government systems, i.e., where the county government performs relatively more of the total government spending, exhibit less spatial variation in working-age mortality. In doing so, this study advances the literature by revealing how that *structure* of local government spending—over and about the *amount* of government spending—shapes spatial variation in population health. Moreover, this study is among the first to identify structural drivers of place-based health inequalities at the sub-county level, i.e., across census tracts, the geographic scale at which we observe the greatest variation in health outcomes.

Future research should explore whether and to what extent other dimensions of local government structure are implicated in the spatial patterning of population health outcomes. Although not the focus of our study, we did find some evidence that the number of local governments in an area is associated with the amount of spatial variation in mortality among those aged 55–64 (see Appendix Table 5; this suggests the degree of local government fragmentation—the number and distribution of general-purpose governments, school districts, and special districts—may also influence spatial variation in health outcomes (see Hutson et al., 2012; Kim & Bruckner, 2016). Additionally, differences in the fiscal powers and capacities of local governments warrants further investigation. Do population health outcomes improve when local governments have greater fiscal autonomy, such as expanded legal authority and administrative capacity to levy taxes and allocate public investments?

Our findings align with broader evidence that local government spending is a critical driver of health outcomes, yet they also reveal that not only the amount but the location and structure of spending matter. Such insights carry important policy implications for federated systems like the United States, where subnational governments operate on unequal tax bases to finance public investments. Achieving equity across places often necessitates either substantial fiscal redistribution—where higher-level governments provide transfers to offset local disparities in fiscal capacity and social need, as is the case in most other wealthy federal democracies such as Germany, Canada and Australia; or the centralization of public finance at a larger spatial scale, thus reducing the influence of local resource inequalities. Although much scholarly attention has centered on the redistributive potential of fiscal transfers—for example, in school finance reforms—the potential equalizing impact of fiscal centralization remains an underexamined yet promising strategy for addressing place-based health disparities. By considering both the amount and location of spending that affects health, policymakers may more effectively target the structural roots of place-based inequalities.

## CRediT authorship contribution statement

**Rourke O'Brien:** Writing – review & editing, Writing – original draft, Supervision, Project administration, Methodology, Funding acquisition, Formal analysis, Data curation, Conceptualization. **Manuel Schechtl:** Writing – review & editing, Project administration, Methodology, Formal analysis, Data curation, Conceptualization. **Robert Manduca:** Writing – review & editing, Methodology, Conceptualization. **Atheendar Venkataramani:** Writing – review & editing, Methodology, Formal analysis, Conceptualization.

## Ethical statement

This analysis only used secondary data that is made publicly available by the U.S. Census Bureau.

## Funding

Research reported in this publication was supported by the National Institute On Aging of the National Institutes of Health under Award Number R24AG045061. The content is solely the responsibility of the authors and does not necessarily represent the official views of the National Institutes of Health.

## Declaration of competing interest

The authors declare that they have no known competing financial interests or personal relationships that could have appeared to influence the work reported in this paper.

## Data Availability

Data will be made available on request.
