# Supplementary material for: Local government expenditure centralization and spatial variation in working-age mortality
Source: SSM Popul Health. 2025 Mar 27;30:101791. doi: 10.1016/j.ssmph.2025.101791 (PMC11997379; doi:10.1016/j.ssmph.2025.101791)
Supplement: Multimedia component 1 [file mmc1.docx]

**Appendix Tables and Figures**

**Table 1** Descriptive statistics by county size

|  | **Small counties**  (< 20,000) | | | **Medium counties**  (20,000-99,999) | | | **Large counties**  (>100,000) | | |
| --- | --- | --- | --- | --- | --- | --- | --- | --- | --- |
|  | mean | min | max | mean | min | max | mean | min | max |
| Expenditure centralization | 0.35 | 0.00 | 1.00 | 0.34 | 0.00 | 1.00 | 0.30 | 0.00 | 1.00 |
| Spatial variation (Gini) in mortality [25-34] | 0.18 | 0.00 | 0.52 | 0.25 | 0.02 | 0.49 | 0.29 | 0.14 | 0.41 |
| Spatial variation (Gini) in mortality [35-44] | 0.18 | 0.00 | 0.50 | 0.23 | 0.03 | 0.54 | 0.30 | 0.16 | 0.46 |
| Spatial variation (Gini) in mortality [45-54] | 0.14 | 0.00 | 0.52 | 0.19 | 0.01 | 0.40 | 0.26 | 0.09 | 0.42 |
| Spatial variation (Gini) in mortality [55-64] | 0.12 | 0.00 | 0.40 | 0.16 | 0.01 | 0.39 | 0.23 | 0.10 | 0.35 |
| Number of Census tracts | 3 | 2 | 17 | 10 | 3 | 36 | 98 | 17 | 2340 |
| Observations | 1068 | | | 1257 | | | 546 | | |

**Figure 1** Within-county spatial variation (Gini) in mortality (25-34)


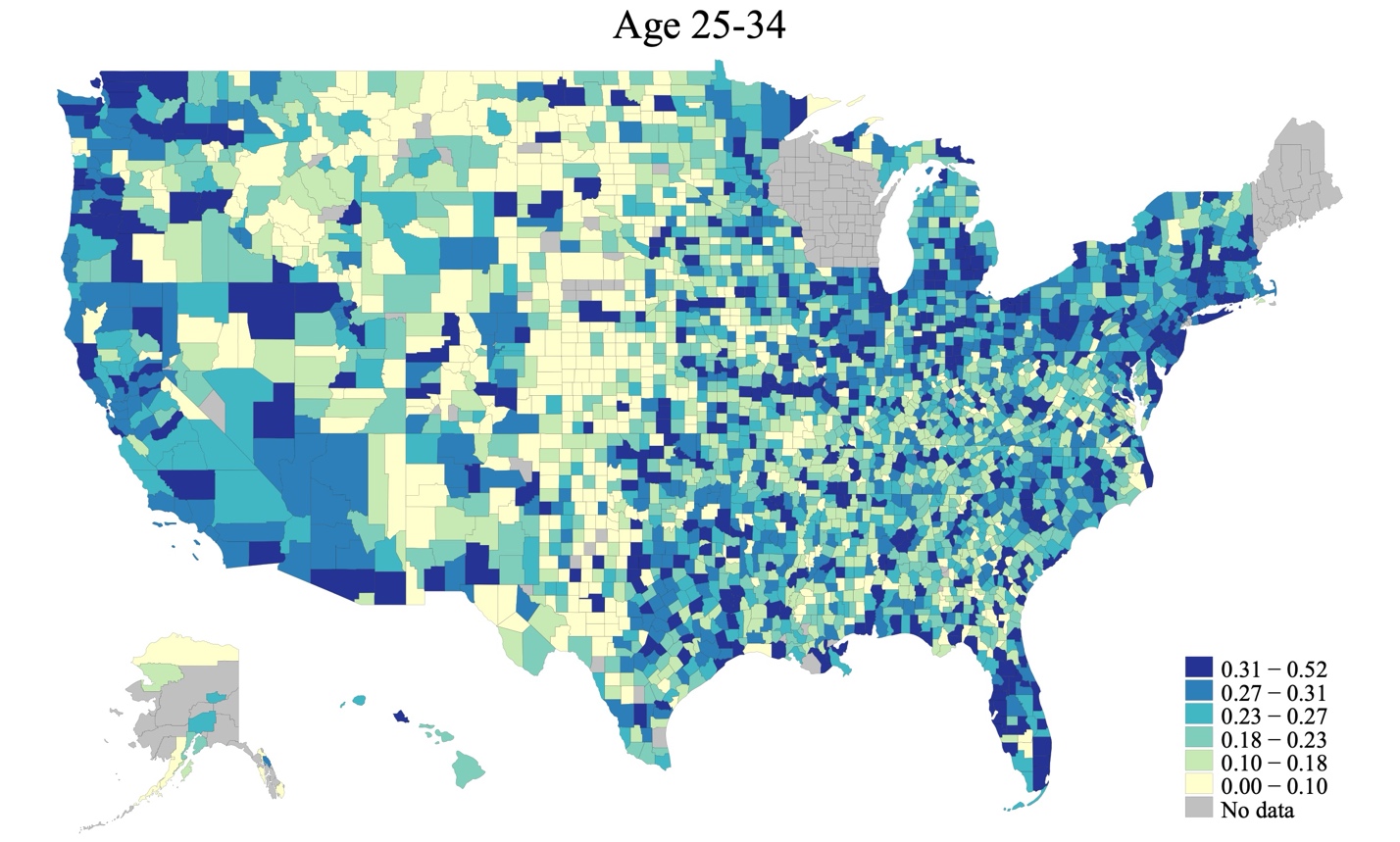


**Figure 2** Within-county spatial variation (Gini) in mortality (35-44)


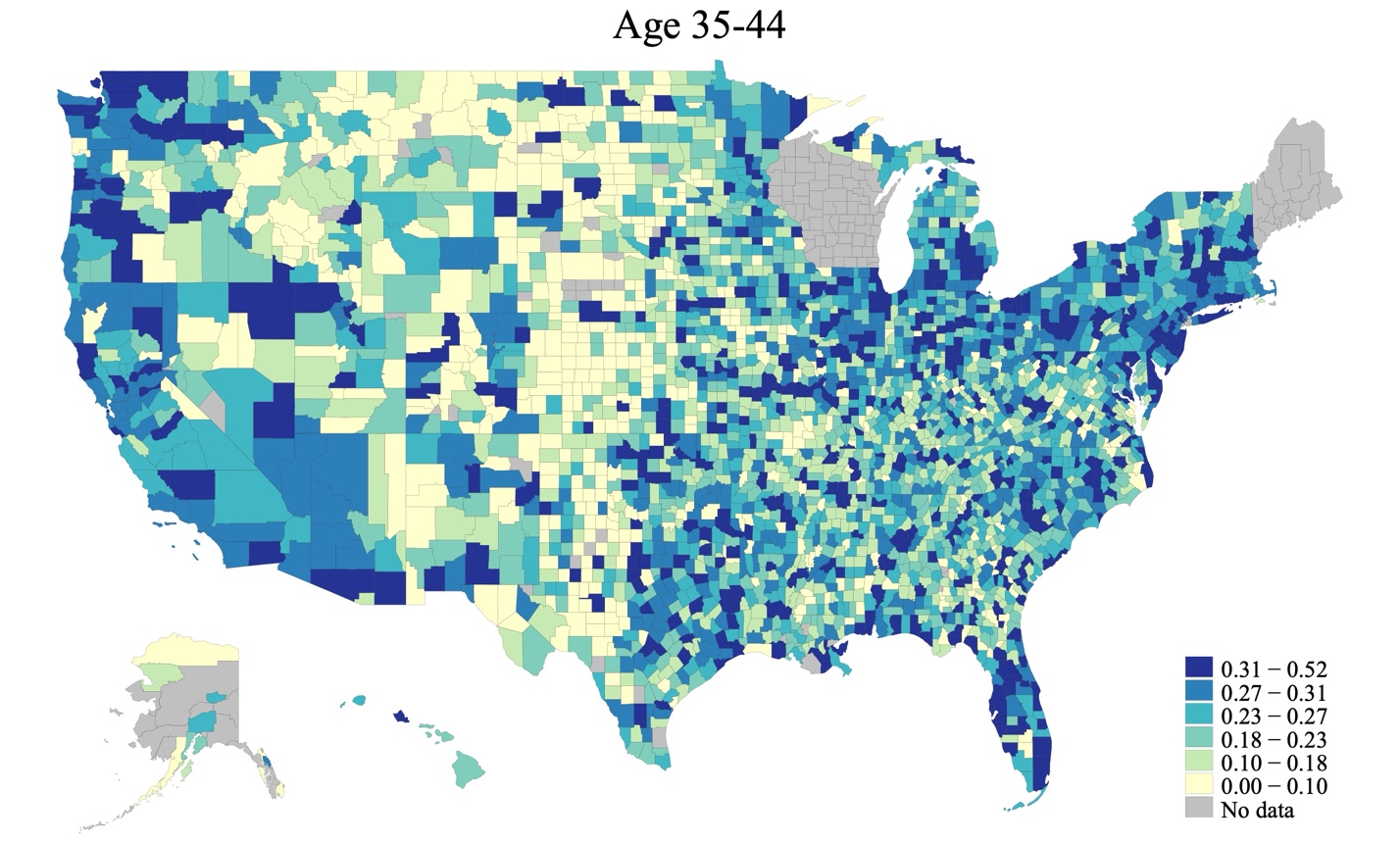


**Figure 3** Within-county spatial variation (Gini) in mortality (45-54)


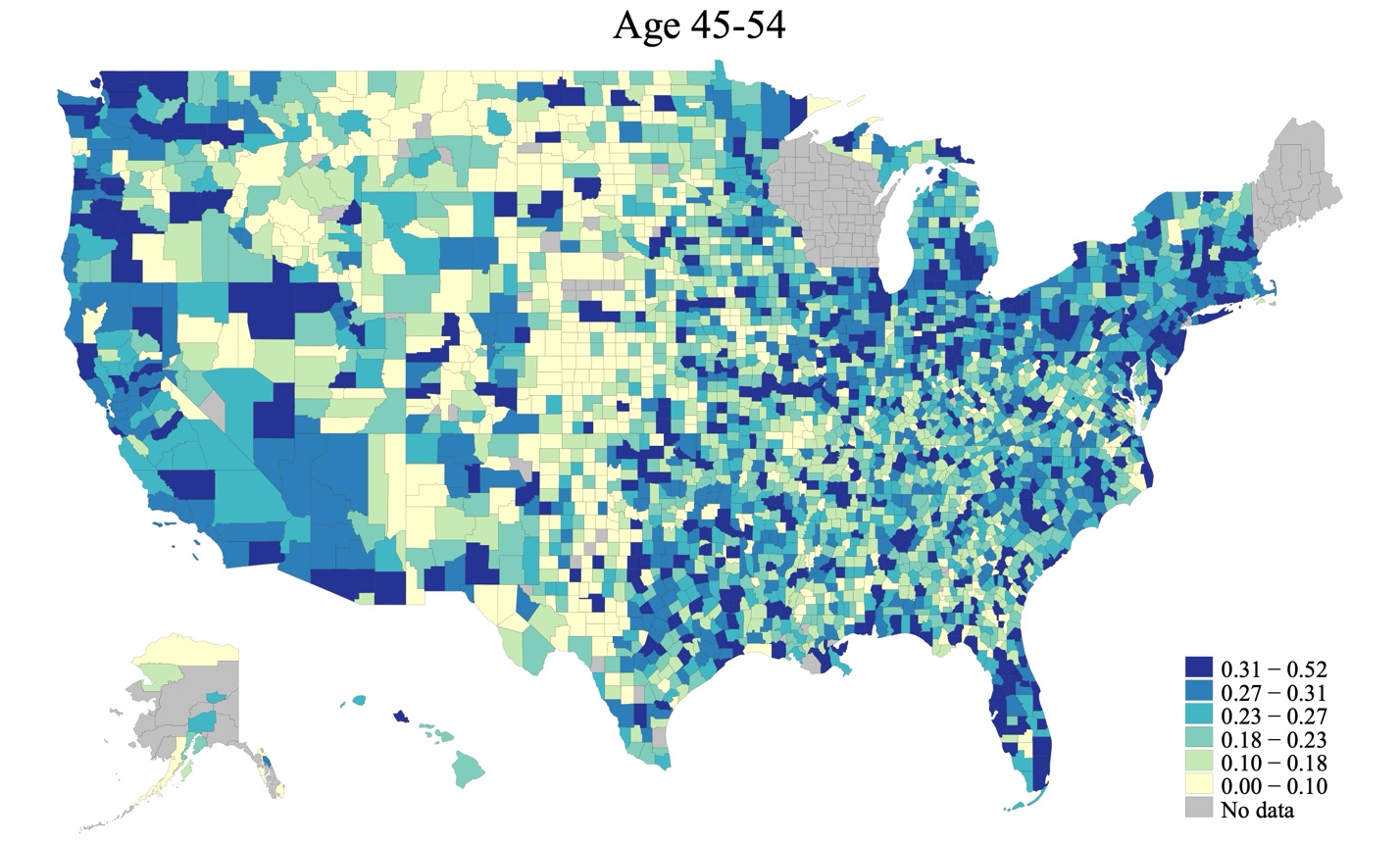


**Figure 4** Within-county spatial variation (Gini) in mortality (55-64)


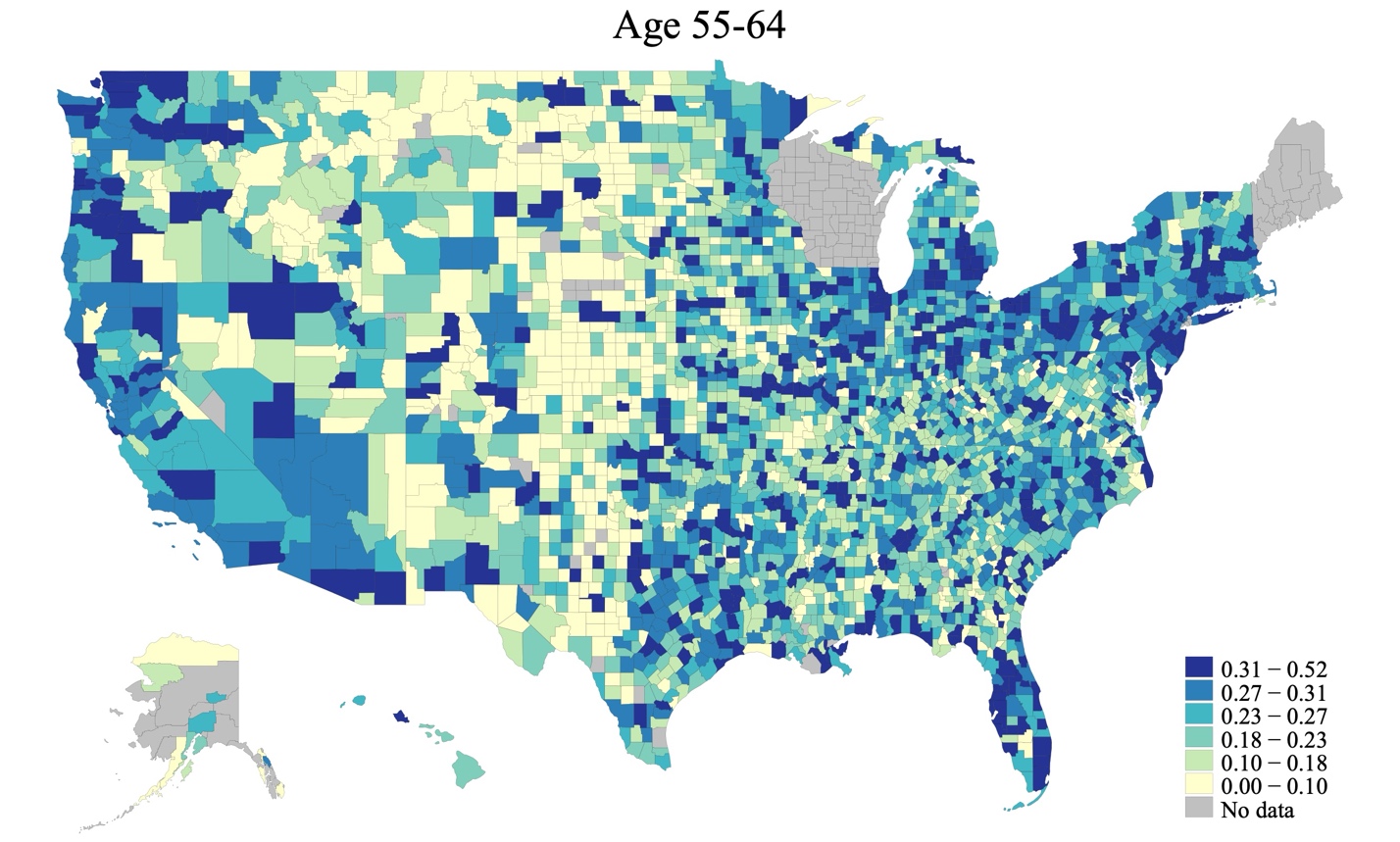


**Figure 5** County-level expenditure centralization 1977 vs. 2007


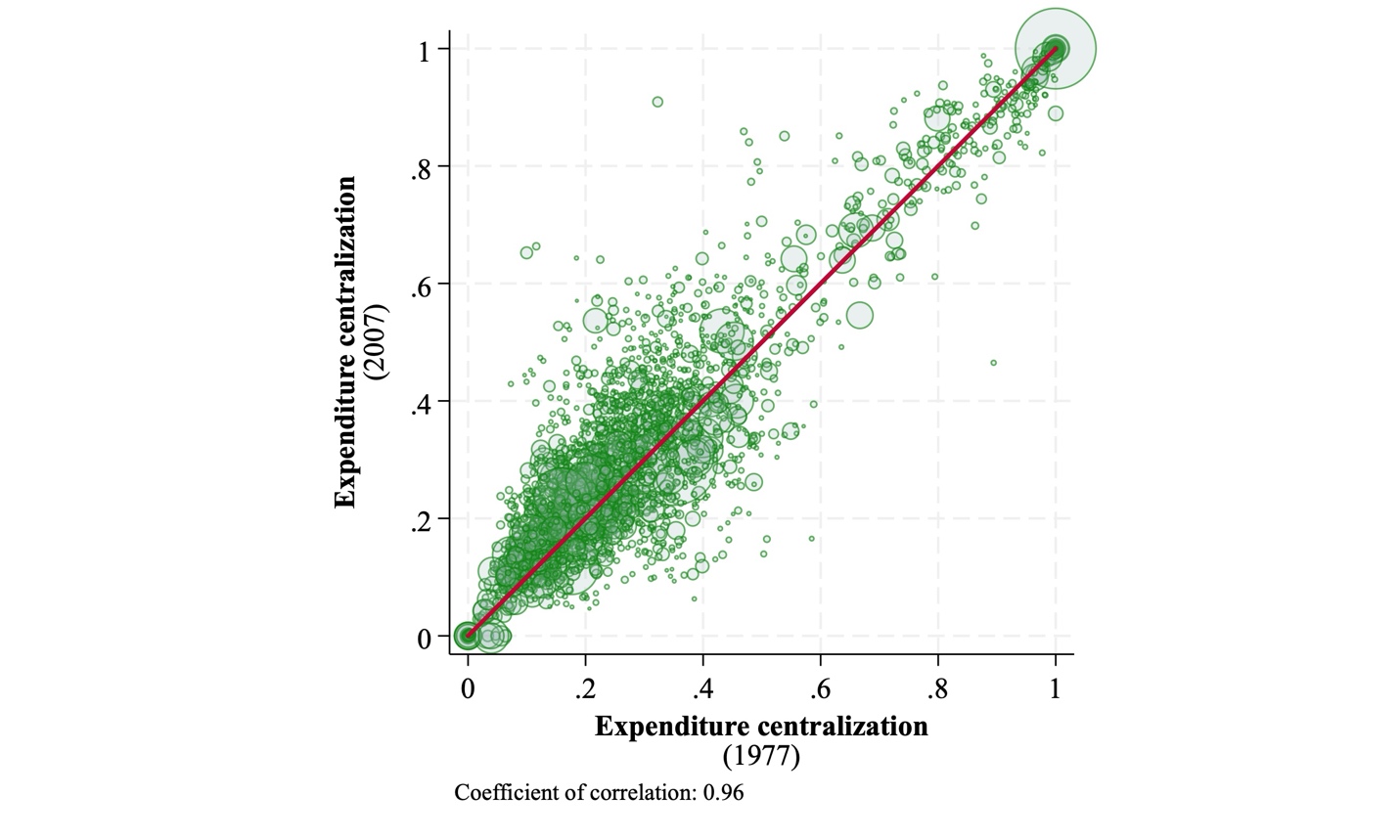


**Figure 6** Coefficients from models estimating relationship between local government expenditure centralization and spatial variation in mortality by 10-year age groups (incl. special district spending)


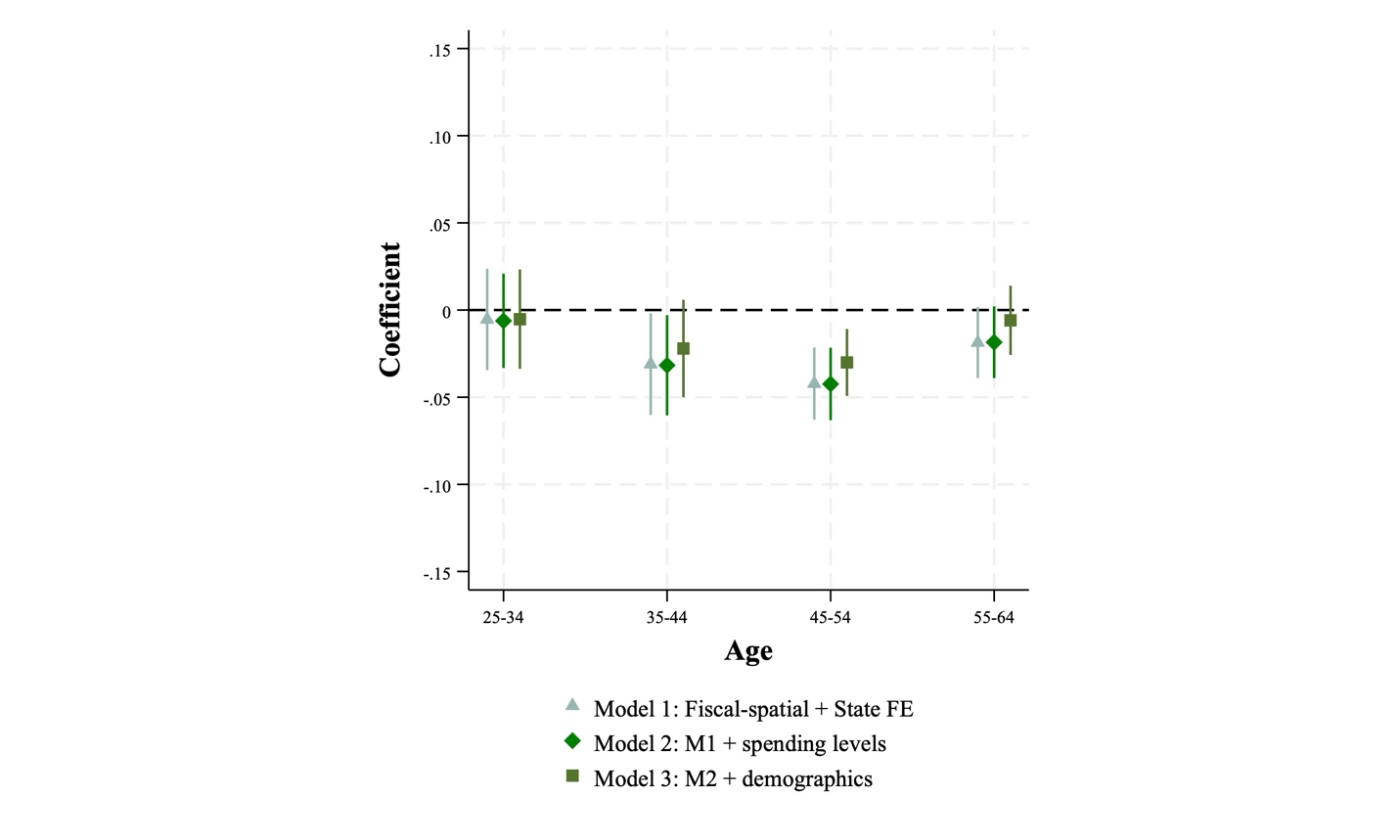


**Table 2** OLS Models Predicting County Area Gini in mortality (25-34)

|  | (1) | (2) | (3) |
| --- | --- | --- | --- |
| VARIABLES | Model 1 | Model 2 | Model 3 |
|  |  |  |  |
| Expenditure centralization | -0.006 | -0.002 | -0.003 |
|  | (0.017) | (0.016) | (0.017) |
| N general purpose governments [log] | 0.001 | 0.001 | 0.001 |
|  | (0.003) | (0.003) | (0.003) |
| N school districts [log] | 0.004 | 0.005 | 0.004 |
|  | (0.005) | (0.005) | (0.005) |
| Population [log] | 0.040*** | 0.038*** | 0.043*** |
|  | (0.008) | (0.008) | (0.009) |
| N census tracts [log] | -0.007 | -0.004 | -0.016 |
|  | (0.009) | (0.009) | (0.010) |
| Land area [log] | 0.001 | 0.001 | 0.001 |
|  | (0.002) | (0.002) | (0.002) |
| Total expenditure [per capita] |  | -0.013 | -0.012 |
|  |  | (0.008) | (0.009) |
| Average income [log] |  |  | -0.031 |
|  |  |  | (0.023) |
| Poverty |  |  | -0.091 |
|  |  |  | (0.071) |
| Migration |  |  | 0.000 |
|  |  |  | (0.000) |
| Share Black |  |  | 0.046** |
|  |  |  | (0.015) |
| College [share] |  |  | -0.015 |
|  |  |  | (0.038) |
| Age 65+ [share] |  |  | 0.035 |
|  |  |  | (0.079) |
| Income inequality [Gini] |  |  | 0.150* |
|  |  |  | (0.065) |
| Poverty segregation [CoV] |  |  | -0.009 |
|  |  |  | (0.012) |
| Racial segregation [CoV] |  |  | 0.014*** |
|  |  |  | (0.004) |
| Constant | -0.189** | -0.154** | 0.129 |
|  | (0.056) | (0.056) | (0.254) |
|  |  |  |  |
| Observations | 2,783 | 2,783 | 2,783 |
| R-squared | 0.291 | 0.292 | 0.301 |

Robust standard errors in parentheses

*** p<0.001, ** p<0.01, * p<0.05

**Table 3** OLS Models Predicting County Area Gini in mortality (35-44)

|  | (1) | (2) | (3) |
| --- | --- | --- | --- |
| VARIABLES | Model 1 | Model 2 | Model 3 |
|  |  |  |  |
| Expenditure centralization | -0.047*** | -0.045*** | -0.035** |
|  | (0.012) | (0.012) | (0.012) |
| N general purpose governments [log] | 0.000 | 0.000 | 0.003 |
|  | (0.004) | (0.004) | (0.004) |
| N school districts [log] | -0.001 | -0.001 | -0.001 |
|  | (0.005) | (0.005) | (0.005) |
| Population [log] | 0.003 | 0.003 | 0.007 |
|  | (0.008) | (0.008) | (0.009) |
| N census tracts [log] | 0.039*** | 0.040*** | 0.020* |
|  | (0.009) | (0.009) | (0.009) |
| Land area [log] | -0.004 | -0.004* | -0.002 |
|  | (0.002) | (0.002) | (0.002) |
| Total expenditure [per capita] |  | -0.005 | -0.011 |
|  |  | (0.008) | (0.008) |
| Average income [log] |  |  | -0.022 |
|  |  |  | (0.021) |
| Poverty |  |  | -0.078 |
|  |  |  | (0.085) |
| Migration |  |  | -0.000 |
|  |  |  | (0.000) |
| Share Black |  |  | 0.021 |
|  |  |  | (0.017) |
| College [share] |  |  | 0.038 |
|  |  |  | (0.032) |
| Age 65+ [share] |  |  | 0.047 |
|  |  |  | (0.064) |
| Income inequality [Gini] |  |  | 0.235*** |
|  |  |  | (0.048) |
| Poverty segregation [CoV] |  |  | 0.020 |
|  |  |  | (0.012) |
| Racial segregation [CoV] |  |  | 0.009* |
|  |  |  | (0.004) |
| Constant | 0.116 | 0.129* | 0.323 |
|  | (0.060) | (0.059) | (0.232) |
|  |  |  |  |
| Observations | 2,783 | 2,783 | 2,783 |
| R-squared | 0.329 | 0.329 | 0.344 |

Robust standard errors in parentheses

*** p<0.001, ** p<0.01, * p<0.05

**Table 4** OLS Models Predicting County Area Gini in mortality (45-54)

|  | (1) | (2) | (3) |
| --- | --- | --- | --- |
| VARIABLES | Model 1 | Model 2 | Model 3 |
|  |  |  |  |
| Expenditure centralization | -0.048*** | -0.047*** | -0.034*** |
|  | (0.009) | (0.010) | (0.009) |
| N general purpose governments [log] | -0.005 | -0.005 | -0.002 |
|  | (0.004) | (0.004) | (0.004) |
| N school districts [log] | 0.007 | 0.007 | 0.008 |
|  | (0.004) | (0.004) | (0.004) |
| Population [log] | -0.016 | -0.016 | -0.012 |
|  | (0.010) | (0.010) | (0.010) |
| N census tracts [log] | 0.060*** | 0.061*** | 0.039** |
|  | (0.011) | (0.011) | (0.012) |
| Land area [log] | -0.003 | -0.004 | -0.002 |
|  | (0.004) | (0.004) | (0.003) |
| Total expenditure [per capita] |  | -0.001 | -0.008 |
|  |  | (0.007) | (0.006) |
| Average income [log] |  |  | -0.022 |
|  |  |  | (0.016) |
| Poverty |  |  | -0.064 |
|  |  |  | (0.050) |
| Migration |  |  | -0.000* |
|  |  |  | (0.000) |
| Share Black |  |  | -0.004 |
|  |  |  | (0.010) |
| College [share] |  |  | 0.044 |
|  |  |  | (0.034) |
| Age 65+ [share] |  |  | 0.058 |
|  |  |  | (0.064) |
| Income inequality [Gini] |  |  | 0.244*** |
|  |  |  | (0.046) |
| Poverty segregation [CoV] |  |  | 0.031* |
|  |  |  | (0.012) |
| Racial segregation [CoV] |  |  | 0.007* |
|  |  |  | (0.003) |
| Constant | 0.214** | 0.217** | 0.405* |
|  | (0.077) | (0.078) | (0.189) |
|  |  |  |  |
| Observations | 2,783 | 2,783 | 2,783 |
| R-squared | 0.388 | 0.388 | 0.409 |

Robust standard errors in parentheses

*** p<0.001, ** p<0.01, * p<0.05

**Table 5** OLS Models Predicting County Area Gini in mortality (55-64)

|  | (1) | (2) | (3) |
| --- | --- | --- | --- |
| VARIABLES | Model 1 | Model 2 | Model 3 |
|  |  |  |  |
| Expenditure centralization | -0.019 | -0.020* | -0.006 |
|  | (0.009) | (0.009) | (0.009) |
| N general purpose governments [log] | -0.006* | -0.006* | -0.003 |
|  | (0.003) | (0.003) | (0.003) |
| N school districts [log] | 0.001 | 0.001 | 0.002 |
|  | (0.003) | (0.003) | (0.003) |
| Population [log] | -0.001 | -0.000 | 0.005 |
|  | (0.005) | (0.005) | (0.005) |
| N census tracts [log] | 0.043*** | 0.042*** | 0.018** |
|  | (0.006) | (0.006) | (0.006) |
| Land area [log] | -0.001 | -0.001 | 0.000 |
|  | (0.003) | (0.003) | (0.002) |
| Total expenditure [per capita] |  | 0.003 | -0.004 |
|  |  | (0.007) | (0.006) |
| Average income [log] |  |  | -0.021 |
|  |  |  | (0.013) |
| Poverty |  |  | -0.049 |
|  |  |  | (0.043) |
| Migration |  |  | -0.000 |
|  |  |  | (0.000) |
| Share Black |  |  | -0.003 |
|  |  |  | (0.009) |
| College [share] |  |  | 0.035 |
|  |  |  | (0.019) |
| Age 65+ [share] |  |  | 0.076 |
|  |  |  | (0.040) |
| Income inequality [Gini] |  |  | 0.314*** |
|  |  |  | (0.049) |
| Poverty segregation [CoV] |  |  | 0.021 |
|  |  |  | (0.012) |
| Racial segregation [CoV] |  |  | 0.008** |
|  |  |  | (0.003) |
| Constant | 0.062 | 0.053 | 0.220 |
|  | (0.043) | (0.042) | (0.147) |
|  |  |  |  |
| Observations | 2,783 | 2,783 | 2,783 |
| R-squared | 0.423 | 0.423 | 0.458 |

Robust standard errors in parentheses

*** p<0.001, ** p<0.01, * p<0.05

**Table 6** OLS Models Predicting County Area Gini in mortality (adjusting for average mortality, 25-34)

|  | (1) | (2) | (3) |
| --- | --- | --- | --- |
| VARIABLES | Model 1 | Model 2 | Model 3 |
|  |  |  |  |
| Expenditure centralization | -0.007 | -0.003 | -0.003 |
|  | (0.017) | (0.016) | (0.017) |
| Average mortality [25-34] | 0.611 | 0.544 | 0.347 |
|  | (0.332) | (0.320) | (0.346) |
| N general purpose governments [log] | 0.001 | 0.001 | 0.001 |
|  | (0.003) | (0.003) | (0.003) |
| N school districts [log] | 0.004 | 0.005 | 0.004 |
|  | (0.005) | (0.005) | (0.005) |
| Population [log] | 0.042*** | 0.039*** | 0.043*** |
|  | (0.008) | (0.008) | (0.009) |
| N census tracts [log] | -0.008 | -0.006 | -0.016 |
|  | (0.009) | (0.010) | (0.010) |
| Land area [log] | 0.008 | 0.006 | 0.006 |
|  | (0.008) | (0.008) | (0.009) |
| Total expenditure [per capita] |  | -0.011 | -0.011 |
|  |  | (0.008) | (0.009) |
| Average income [log] |  |  | -0.031 |
|  |  |  | (0.023) |
| Poverty |  |  | -0.095 |
|  |  |  | (0.070) |
| Migration |  |  | 0.000 |
|  |  |  | (0.000) |
| Share Black |  |  | 0.044** |
|  |  |  | (0.016) |
| College [share] |  |  | -0.009 |
|  |  |  | (0.038) |
| Age 65+ [share] |  |  | 0.028 |
|  |  |  | (0.080) |
| Income inequality [Gini] |  |  | 0.150* |
|  |  |  | (0.065) |
| Poverty segregation [CoV] |  |  | -0.008 |
|  |  |  | (0.012) |
| Racial segregation [CoV] |  |  | 0.014*** |
|  |  |  | (0.004) |
| Constant | -0.222*** | -0.185** | 0.119 |
|  | (0.061) | (0.061) | (0.253) |
|  |  |  |  |
| Observations | 2,783 | 2,783 | 2,783 |
| R-squared | 0.292 | 0.293 | 0.301 |

Robust standard errors in parentheses

*** p<0.001, ** p<0.01, * p<0.05

**Table 7** OLS Models Predicting County Area Gini in mortality (adjusting for average mortality, 35-44)

|  | (1) | (2) | (3) |
| --- | --- | --- | --- |
| VARIABLES | Model 1 | Model 2 | Model 3 |
|  |  |  |  |
| Expenditure centralization | -0.047*** | -0.045*** | -0.036** |
|  | (0.012) | (0.012) | (0.012) |
| Average mortality [35-44] | -0.100 | -0.115 | 0.007 |
|  | (0.390) | (0.387) | (0.479) |
| N general purpose governments [log] | -0.000 | -0.000 | 0.002 |
|  | (0.004) | (0.004) | (0.004) |
| N school districts [log] | -0.002 | -0.001 | -0.001 |
|  | (0.005) | (0.005) | (0.005) |
| Population [log] | 0.003 | 0.002 | 0.007 |
|  | (0.009) | (0.009) | (0.009) |
| N census tracts [log] | 0.039*** | 0.040*** | 0.020* |
|  | (0.010) | (0.010) | (0.010) |
| Land area [log] | -0.014 | -0.015 | -0.007 |
|  | (0.009) | (0.008) | (0.008) |
| Total expenditure [per capita] |  | -0.005 | -0.011 |
|  |  | (0.008) | (0.008) |
| Average income [log] |  |  | -0.022 |
|  |  |  | (0.021) |
| Poverty |  |  | -0.079 |
|  |  |  | (0.089) |
| Migration |  |  | -0.000 |
|  |  |  | (0.000) |
| Share Black |  |  | 0.021 |
|  |  |  | (0.018) |
| College [share] |  |  | 0.039 |
|  |  |  | (0.033) |
| Age 65+ [share] |  |  | 0.048 |
|  |  |  | (0.066) |
| Income inequality [Gini] |  |  | 0.235*** |
|  |  |  | (0.047) |
| Poverty segregation [CoV] |  |  | 0.020 |
|  |  |  | (0.012) |
| Racial segregation [CoV] |  |  | 0.009* |
|  |  |  | (0.004) |
| Constant | 0.123 | 0.139 | 0.316 |
|  | (0.073) | (0.073) | (0.228) |
|  |  |  |  |
| Observations | 2,783 | 2,783 | 2,783 |
| R-squared | 0.329 | 0.329 | 0.344 |

Robust standard errors in parentheses

*** p<0.001, ** p<0.01, * p<0.05

**Table 8** OLS Models Predicting County Area Gini in mortality (adjusting for average mortality, 45-54)

|  | (1) | (2) | (3) |
| --- | --- | --- | --- |
| VARIABLES | Model 1 | Model 2 | Model 3 |
|  |  |  |  |
| Expenditure centralization | -0.046*** | -0.046*** | -0.033*** |
|  | (0.009) | (0.010) | (0.009) |
| Average mortality [45-54] | 0.526* | 0.529* | 0.911*** |
|  | (0.211) | (0.211) | (0.236) |
| N general purpose governments [log] | -0.004 | -0.004 | -0.000 |
|  | (0.004) | (0.004) | (0.003) |
| N school districts [log] | 0.006 | 0.005 | 0.007 |
|  | (0.004) | (0.004) | (0.004) |
| Population [log] | -0.013 | -0.013 | -0.013 |
|  | (0.010) | (0.010) | (0.010) |
| N census tracts [log] | 0.059*** | 0.058*** | 0.039** |
|  | (0.011) | (0.012) | (0.012) |
| Land area [log] | -0.026 | -0.026 | -0.010 |
|  | (0.015) | (0.014) | (0.015) |
| Total expenditure [per capita] |  | 0.001 | -0.007 |
|  |  | (0.007) | (0.006) |
| Average income [log] |  |  | -0.010 |
|  |  |  | (0.016) |
| Poverty |  |  | -0.111* |
|  |  |  | (0.052) |
| Migration |  |  | -0.000** |
|  |  |  | (0.000) |
| Share Black |  |  | -0.012 |
|  |  |  | (0.012) |
| College [share] |  |  | 0.087* |
|  |  |  | (0.037) |
| Age 65+ [share] |  |  | 0.027 |
|  |  |  | (0.057) |
| Income inequality [Gini] |  |  | 0.235*** |
|  |  |  | (0.048) |
| Poverty segregation [CoV] |  |  | 0.032* |
|  |  |  | (0.012) |
| Racial segregation [CoV] |  |  | 0.006* |
|  |  |  | (0.003) |
| Constant | 0.184* | 0.180* | 0.251 |
|  | (0.084) | (0.087) | (0.196) |
|  |  |  |  |
| Observations | 2,783 | 2,783 | 2,783 |
| R-squared | 0.394 | 0.394 | 0.420 |

Robust standard errors in parentheses

*** p<0.001, ** p<0.01, * p<0.05

**Table 9** OLS Models Predicting County Area Gini in mortality (adjusting for average mortality, 55-64)

|  | (1) | (2) | (3) |
| --- | --- | --- | --- |
| VARIABLES | Model 1 | Model 2 | Model 3 |
|  |  |  |  |
| Expenditure centralization | -0.015 | -0.016 | -0.003 |
|  | (0.009) | (0.009) | (0.009) |
| Average mortality [55-64] | 0.359** | 0.364** | 0.629*** |
|  | (0.110) | (0.108) | (0.138) |
| N general purpose governments [log] | -0.006 | -0.006 | -0.002 |
|  | (0.003) | (0.003) | (0.003) |
| N school districts [log] | -0.000 | -0.001 | 0.001 |
|  | (0.003) | (0.003) | (0.003) |
| Population [log] | 0.001 | 0.002 | 0.002 |
|  | (0.005) | (0.005) | (0.005) |
| N census tracts [log] | 0.042*** | 0.041*** | 0.019** |
|  | (0.006) | (0.006) | (0.005) |
| Land area [log] | -0.008 | -0.007 | 0.013 |
|  | (0.011) | (0.011) | (0.011) |
| Total expenditure [per capita] |  | 0.006 | -0.004 |
|  |  | (0.007) | (0.007) |
| Average income [log] |  |  | -0.003 |
|  |  |  | (0.015) |
| Poverty |  |  | -0.077 |
|  |  |  | (0.042) |
| Migration |  |  | -0.000 |
|  |  |  | (0.000) |
| Share Black |  |  | -0.017 |
|  |  |  | (0.011) |
| College [share] |  |  | 0.093*** |
|  |  |  | (0.023) |
| Age 65+ [share] |  |  | 0.078* |
|  |  |  | (0.032) |
| Income inequality [Gini] |  |  | 0.302*** |
|  |  |  | (0.050) |
| Poverty segregation [CoV] |  |  | 0.021 |
|  |  |  | (0.011) |
| Racial segregation [CoV] |  |  | 0.006* |
|  |  |  | (0.003) |
| Constant | 0.012 | -0.005 | -0.039 |
|  | (0.044) | (0.039) | (0.160) |
|  |  |  |  |
| Observations | 2,783 | 2,783 | 2,783 |
| R-squared | 0.431 | 0.431 | 0.473 |

Robust standard errors in parentheses

*** p<0.001, ** p<0.01, * p<0.05

**Table 10** OLS Models Predicting County Area Gini in mortality (weighted, 25-34)

|  | (1) | (2) | (3) |
| --- | --- | --- | --- |
| VARIABLES | Model 1 | Model 2 | Model 3 |
|  |  |  |  |
| Expenditure centralization | -0.023 | -0.016 | -0.014 |
|  | (0.018) | (0.015) | (0.015) |
| N general purpose governments [log] | -0.001 | -0.001 | 0.000 |
|  | (0.003) | (0.003) | (0.003) |
| N school districts [log] | 0.007 | 0.008 | 0.008 |
|  | (0.005) | (0.005) | (0.005) |
| N census tracts [log] | 0.035*** | 0.036*** | 0.026*** |
|  | (0.002) | (0.002) | (0.003) |
| Land area [log] | 0.010 | 0.005 | 0.007 |
|  | (0.008) | (0.008) | (0.008) |
| Total expenditure [per capita] |  | -0.021* | -0.021* |
|  |  | (0.008) | (0.008) |
| Average income [log] |  |  | -0.033 |
|  |  |  | (0.022) |
| Poverty |  |  | -0.113 |
|  |  |  | (0.067) |
| Migration |  |  | 0.001** |
|  |  |  | (0.000) |
| Share Black |  |  | 0.039* |
|  |  |  | (0.015) |
| College [share] |  |  | -0.006 |
|  |  |  | (0.036) |
| Age 65+ [share] |  |  | -0.103 |
|  |  |  | (0.072) |
| Income inequality [Gini] |  |  | 0.161* |
|  |  |  | (0.063) |
| Poverty segregation [CoV] |  |  | -0.010 |
|  |  |  | (0.011) |
| Racial segregation [CoV] |  |  | 0.015*** |
|  |  |  | (0.004) |
| Constant | 0.123*** | 0.156*** | 0.537* |
|  | (0.018) | (0.021) | (0.251) |
|  |  |  |  |
| Observations | 2,783 | 2,783 | 2,783 |
| R-squared | 0.284 | 0.287 | 0.296 |

Robust standard errors in parentheses

*** p<0.001, ** p<0.01, * p<0.05

**Table 11** OLS Models Predicting County Area Gini in mortality (weighted, 35-44)

|  | (1) | (2) | (3) |
| --- | --- | --- | --- |
| VARIABLES | Model 1 | Model 2 | Model 3 |
|  |  |  |  |
| Expenditure centralization | -0.047*** | -0.046*** | -0.036** |
|  | (0.013) | (0.012) | (0.012) |
| N general purpose governments [log] | -0.000 | -0.000 | 0.002 |
|  | (0.004) | (0.004) | (0.004) |
| N school districts [log] | -0.002 | -0.002 | -0.001 |
|  | (0.005) | (0.005) | (0.005) |
| N census tracts [log] | 0.041*** | 0.041*** | 0.024*** |
|  | (0.002) | (0.002) | (0.003) |
| Land area [log] | -0.014 | -0.015 | -0.005 |
|  | (0.009) | (0.008) | (0.008) |
| Total expenditure [per capita] |  | -0.004 | -0.011 |
|  |  | (0.008) | (0.007) |
| Average income [log] |  |  | -0.028 |
|  |  |  | (0.022) |
| Poverty |  |  | -0.102 |
|  |  |  | (0.084) |
| Migration |  |  | -0.000 |
|  |  |  | (0.000) |
| Share Black |  |  | 0.024 |
|  |  |  | (0.016) |
| College [share] |  |  | 0.042 |
|  |  |  | (0.031) |
| Age 65+ [share] |  |  | 0.029 |
|  |  |  | (0.056) |
| Income inequality [Gini] |  |  | 0.255*** |
|  |  |  | (0.046) |
| Poverty segregation [CoV] |  |  | 0.021 |
|  |  |  | (0.011) |
| Racial segregation [CoV] |  |  | 0.010* |
|  |  |  | (0.004) |
| Constant | 0.149*** | 0.156*** | 0.445 |
|  | (0.020) | (0.021) | (0.243) |
|  |  |  |  |
| Observations | 2,783 | 2,783 | 2,783 |
| R-squared | 0.345 | 0.345 | 0.363 |

Robust standard errors in parentheses

*** p<0.001, ** p<0.01, * p<0.05

**Table 12** OLS Models Predicting County Area Gini in mortality (weighted, 45-54)

|  | (1) | (2) | (3) |
| --- | --- | --- | --- |
| VARIABLES | Model 1 | Model 2 | Model 3 |
|  |  |  |  |
| Expenditure centralization | -0.046*** | -0.047*** | -0.034*** |
|  | (0.010) | (0.010) | (0.009) |
| N general purpose governments [log] | -0.005 | -0.005 | -0.002 |
|  | (0.004) | (0.004) | (0.003) |
| N school districts [log] | 0.004 | 0.004 | 0.005 |
|  | (0.004) | (0.004) | (0.004) |
| N census tracts [log] | 0.042*** | 0.042*** | 0.025*** |
|  | (0.002) | (0.002) | (0.003) |
| Land area [log] | -0.022 | -0.021 | -0.013 |
|  | (0.016) | (0.015) | (0.014) |
| Total expenditure [per capita] |  | 0.003 | -0.005 |
|  |  | (0.007) | (0.006) |
| Average income [log] |  |  | -0.028 |
|  |  |  | (0.015) |
| Poverty |  |  | -0.080 |
|  |  |  | (0.052) |
| Migration |  |  | -0.000** |
|  |  |  | (0.000) |
| Share Black |  |  | 0.001 |
|  |  |  | (0.010) |
| College [share] |  |  | 0.050 |
|  |  |  | (0.032) |
| Age 65+ [share] |  |  | 0.086 |
|  |  |  | (0.063) |
| Income inequality [Gini] |  |  | 0.272*** |
|  |  |  | (0.045) |
| Poverty segregation [CoV] |  |  | 0.033** |
|  |  |  | (0.012) |
| Racial segregation [CoV] |  |  | 0.007* |
|  |  |  | (0.003) |
| Constant | 0.116*** | 0.110*** | 0.392* |
|  | (0.029) | (0.027) | (0.179) |
|  |  |  |  |
| Observations | 2,783 | 2,783 | 2,783 |
| R-squared | 0.410 | 0.410 | 0.437 |

Robust standard errors in parentheses

*** p<0.001, ** p<0.01, * p<0.05

**Table 13** OLS Models Predicting County Area Gini in mortality (weighted, 55-64)

|  | (1) | (2) | (3) |
| --- | --- | --- | --- |
| VARIABLES | Model 1 | Model 2 | Model 3 |
|  |  |  |  |
| Expenditure centralization | -0.022* | -0.024* | -0.010 |
|  | (0.010) | (0.010) | (0.009) |
| N general purpose governments [log] | -0.006* | -0.006* | -0.003 |
|  | (0.003) | (0.003) | (0.003) |
| N school districts [log] | 0.001 | 0.000 | 0.002 |
|  | (0.003) | (0.003) | (0.003) |
| N census tracts [log] | 0.041*** | 0.041*** | 0.021*** |
|  | (0.002) | (0.002) | (0.003) |
| Land area [log] | -0.007 | -0.006 | 0.003 |
|  | (0.011) | (0.010) | (0.009) |
| Total expenditure [per capita] |  | 0.004 | -0.005 |
|  |  | (0.007) | (0.006) |
| Average income [log] |  |  | -0.025 |
|  |  |  | (0.013) |
| Poverty |  |  | -0.063 |
|  |  |  | (0.043) |
| Migration |  |  | -0.000 |
|  |  |  | (0.000) |
| Share Black |  |  | -0.003 |
|  |  |  | (0.009) |
| College [share] |  |  | 0.038* |
|  |  |  | (0.018) |
| Age 65+ [share] |  |  | 0.059 |
|  |  |  | (0.039) |
| Income inequality [Gini] |  |  | 0.329*** |
|  |  |  | (0.048) |
| Poverty segregation [CoV] |  |  | 0.023* |
|  |  |  | (0.011) |
| Racial segregation [CoV] |  |  | 0.007** |
|  |  |  | (0.003) |
| Constant | 0.066** | 0.060** | 0.307* |
|  | (0.020) | (0.020) | (0.150) |
|  |  |  |  |
| Observations | 2,783 | 2,783 | 2,783 |
| R-squared | 0.447 | 0.447 | 0.486 |

Robust standard errors in parentheses

*** p<0.001, ** p<0.01, * p<0.05

**Table 14** OLS Models Predicting County Area Gini in mortality (excl. school district spending, 25-34)

|  | (1) | (2) | (3) |
| --- | --- | --- | --- |
| VARIABLES | Model 1 | Model 2 | Model 3 |
|  |  |  |  |
| Expenditure centralization | -0.019 | -0.020 | -0.016 |
|  | (0.011) | (0.010) | (0.011) |
| N general purpose governments [log] | -0.000 | -0.000 | -0.000 |
|  | (0.003) | (0.003) | (0.003) |
| N school districts [log] | 0.004 | 0.004 | 0.003 |
|  | (0.005) | (0.005) | (0.005) |
| Population [log] | 0.039*** | 0.037*** | 0.042*** |
|  | (0.008) | (0.008) | (0.009) |
| N census tracts [log] | -0.007 | -0.004 | -0.015 |
|  | (0.009) | (0.009) | (0.010) |
| Land area [log] | 0.011 | 0.009 | 0.007 |
|  | (0.009) | (0.008) | (0.009) |
| Total expenditure [per capita] |  | -0.008 | -0.009 |
|  |  | (0.007) | (0.007) |
| Average income [log] |  |  | -0.030 |
|  |  |  | (0.022) |
| Poverty |  |  | -0.090 |
|  |  |  | (0.069) |
| Migration |  |  | 0.000 |
|  |  |  | (0.000) |
| Share Black |  |  | 0.046** |
|  |  |  | (0.015) |
| College [share] |  |  | -0.016 |
|  |  |  | (0.038) |
| Age 65+ [share] |  |  | 0.039 |
|  |  |  | (0.079) |
| Income inequality [Gini] |  |  | 0.142* |
|  |  |  | (0.065) |
| Poverty segregation [CoV] |  |  | -0.009 |
|  |  |  | (0.012) |
| Racial segregation [CoV] |  |  | 0.015*** |
|  |  |  | (0.004) |
| Constant | -0.180** | -0.156** | 0.121 |
|  | (0.059) | (0.055) | (0.251) |
|  |  |  |  |
| Observations | 2,783 | 2,783 | 2,783 |
| R-squared | 0.292 | 0.293 | 0.301 |

Robust standard errors in parentheses

*** p<0.001, ** p<0.01, * p<0.05

**Table 15** OLS Models Predicting County Area Gini in mortality (excl. school district spending, 35-44)

|  | (1) | (2) | (3) |
| --- | --- | --- | --- |
| VARIABLES | Model 1 | Model 2 | Model 3 |
|  |  |  |  |
| Expenditure centralization | -0.038*** | -0.039*** | -0.028** |
|  | (0.009) | (0.009) | (0.009) |
| N general purpose governments [log] | -0.001 | -0.001 | 0.002 |
|  | (0.004) | (0.004) | (0.004) |
| N school districts [log] | -0.001 | -0.001 | -0.001 |
|  | (0.005) | (0.005) | (0.005) |
| Population [log] | 0.004 | 0.002 | 0.006 |
|  | (0.008) | (0.008) | (0.009) |
| N census tracts [log] | 0.038*** | 0.040*** | 0.021* |
|  | (0.010) | (0.009) | (0.009) |
| Land area [log] | -0.013 | -0.015 | -0.007 |
|  | (0.009) | (0.008) | (0.008) |
| Total expenditure [per capita] |  | -0.008 | -0.014* |
|  |  | (0.006) | (0.006) |
| Average income [log] |  |  | -0.020 |
|  |  |  | (0.021) |
| Poverty |  |  | -0.075 |
|  |  |  | (0.085) |
| Migration |  |  | -0.000 |
|  |  |  | (0.000) |
| Share Black |  |  | 0.021 |
|  |  |  | (0.018) |
| College [share] |  |  | 0.036 |
|  |  |  | (0.032) |
| Age 65+ [share] |  |  | 0.047 |
|  |  |  | (0.064) |
| Income inequality [Gini] |  |  | 0.231*** |
|  |  |  | (0.047) |
| Poverty segregation [CoV] |  |  | 0.020 |
|  |  |  | (0.012) |
| Racial segregation [CoV] |  |  | 0.009* |
|  |  |  | (0.004) |
| Constant | 0.125 | 0.150* | 0.308 |
|  | (0.065) | (0.060) | (0.232) |
|  |  |  |  |
| Observations | 2,783 | 2,783 | 2,783 |
| R-squared | 0.330 | 0.330 | 0.344 |

Robust standard errors in parentheses

*** p<0.001, ** p<0.01, * p<0.05

**Table 16** OLS Models Predicting County Area Gini in mortality (excl. school district spending, 45-54)

|  | (1) | (2) | (3) |
| --- | --- | --- | --- |
| VARIABLES | Model 1 | Model 2 | Model 3 |
|  |  |  |  |
| Expenditure centralization | -0.037*** | -0.037*** | -0.025** |
|  | (0.008) | (0.008) | (0.008) |
| N general purpose governments [log] | -0.005 | -0.005 | -0.002 |
|  | (0.004) | (0.004) | (0.003) |
| N school districts [log] | 0.008 | 0.008 | 0.008 |
|  | (0.004) | (0.004) | (0.004) |
| Population [log] | -0.015 | -0.016 | -0.012 |
|  | (0.010) | (0.010) | (0.010) |
| N census tracts [log] | 0.059*** | 0.060*** | 0.040** |
|  | (0.011) | (0.011) | (0.011) |
| Land area [log] | -0.022 | -0.023 | -0.015 |
|  | (0.015) | (0.016) | (0.014) |
| Total expenditure [per capita] |  | -0.004 | -0.009 |
|  |  | (0.005) | (0.005) |
| Average income [log] |  |  | -0.021 |
|  |  |  | (0.016) |
| Poverty |  |  | -0.060 |
|  |  |  | (0.052) |
| Migration |  |  | -0.000* |
|  |  |  | (0.000) |
| Share Black |  |  | -0.006 |
|  |  |  | (0.010) |
| College [share] |  |  | 0.038 |
|  |  |  | (0.034) |
| Age 65+ [share] |  |  | 0.056 |
|  |  |  | (0.064) |
| Income inequality [Gini] |  |  | 0.239*** |
|  |  |  | (0.047) |
| Poverty segregation [CoV] |  |  | 0.031* |
|  |  |  | (0.012) |
| Racial segregation [CoV] |  |  | 0.007* |
|  |  |  | (0.003) |
| Constant | 0.240** | 0.253** | 0.412* |
|  | (0.076) | (0.076) | (0.192) |
|  |  |  |  |
| Observations | 2,783 | 2,783 | 2,783 |
| R-squared | 0.389 | 0.389 | 0.410 |

Robust standard errors in parentheses

*** p<0.001, ** p<0.01, * p<0.05

**Table 17** OLS Models Predicting County Area Gini in mortality (excl. school district spending, 55-64)

|  | (1) | (2) | (3) |
| --- | --- | --- | --- |
| VARIABLES | Model 1 | Model 2 | Model 3 |
|  |  |  |  |
| Expenditure centralization | -0.021*** | -0.020*** | -0.007 |
|  | (0.006) | (0.006) | (0.006) |
| N general purpose governments [log] | -0.007* | -0.007* | -0.003 |
|  | (0.003) | (0.003) | (0.003) |
| N school districts [log] | 0.001 | 0.001 | 0.002 |
|  | (0.003) | (0.003) | (0.003) |
| Population [log] | -0.001 | -0.000 | 0.005 |
|  | (0.005) | (0.005) | (0.005) |
| N census tracts [log] | 0.043*** | 0.042*** | 0.018** |
|  | (0.006) | (0.006) | (0.006) |
| Land area [log] | -0.007 | -0.006 | 0.003 |
|  | (0.011) | (0.011) | (0.010) |
| Total expenditure [per capita] |  | 0.002 | -0.004 |
|  |  | (0.006) | (0.005) |
| Average income [log] |  |  | -0.021 |
|  |  |  | (0.013) |
| Poverty |  |  | -0.048 |
|  |  |  | (0.042) |
| Migration |  |  | -0.000 |
|  |  |  | (0.000) |
| Share Black |  |  | -0.003 |
|  |  |  | (0.009) |
| College [share] |  |  | 0.035 |
|  |  |  | (0.020) |
| Age 65+ [share] |  |  | 0.076 |
|  |  |  | (0.040) |
| Income inequality [Gini] |  |  | 0.312*** |
|  |  |  | (0.048) |
| Poverty segregation [CoV] |  |  | 0.021 |
|  |  |  | (0.012) |
| Racial segregation [CoV] |  |  | 0.008** |
|  |  |  | (0.003) |
| Constant | 0.076 | 0.068 | 0.216 |
|  | (0.044) | (0.041) | (0.147) |
|  |  |  |  |
| Observations | 2,783 | 2,783 | 2,783 |
| R-squared | 0.424 | 0.424 | 0.458 |

Robust standard errors in parentheses

*** p<0.001, ** p<0.01, * p<0.05
